# Psychosocial predictors of post‐natal anxiety and depression: Using Structural Equation Modelling to investigate the relationship between pressure to breastfeed, health care professional support, post‐natal guilt and shame, and post‐natal anxiety and depression within an infant feeding context

**DOI:** 10.1111/mcn.13558

**Published:** 2023-09-26

**Authors:** Leanne Jackson, Vicky Fallon, Joanne A. Harrold, Leonardo De Pascalis

**Affiliations:** ^1^ Department of Psychological Sciences University of Liverpool Liverpool Merseyside UK

**Keywords:** breastfeeding, infant milk formula, maternal mental health, post‐natal care, postpartum, social factors

## Abstract

High perceived pressure to breastfeed and poor perceived quality of health care professional support have been associated with early breastfeeding cessation, guilt, and shame. This is problematic because guilt and shame significantly predict post‐natal anxiety and depression. No previous attempts have been made to provide quantitative evidence for relationships mapped between the post‐natal social context, infant feeding method and post‐natal emotional well‐being. The current study aimed to empirically investigate aforementioned pathways. Structural equation modelling was applied to survey data provided online by 876 mothers. Guilt and shame both significantly predicted anxiety and depression. Poor health care professional support and high pressure to breastfeed increased anxiety and depression, and these effects were explained by indirect pathways through increases in guilt and shame. Formula feeding exclusivity was negatively correlated with post‐natal anxiety symptoms. This finding may be explained by feelings of relief associated with observed infant weight gain and being able to share infant feeding responsibilities others e.g., with one's partner. This relationship was counterbalanced by an indirect pathway where greater formula feeding exclusivity positively predicted guilt, which increased post‐natal anxiety score. While guilt acted as mediator of infant feeding method to increase post‐natal depression and anxiety, shame acted independently of infant feeding method. These identified differences provide empirical support for the transferability of general definitions of guilt (i.e., as remorse for having committed a moral transgression) and shame (i.e., internalisation of transgressive remorse to the self), to an infant feeding context. Recommendations for health care practitioners and the maternal social support network are discussed.

## Key messages

- Guilt and shame are empirically evidenced as distinct, overlapping concepts: guilt is more behaviour‐orientated, while shame is more social context‐orientated.
- Guilt and shame both predict postnatal depression and anxiety, supporting their conceptualisation as transdiagnostic phenomena.
- While guilt acts indirectly through one's social context and infant feeding method to increase depression and anxiety, shame acts independently of infant feeding method. General definitions of guilt and shame are thus deemed transferable to an infant feeding context.
- Recommendations are made to address issues regarding perceived insufficient healthcare professional support and pressure to breastfeed, so to improve postnatal wellbeing and extend any breastfeeding continuation.

## Graphical abstract

The current study aimed to provide quantitative evidence for the relationship between the post‐natal social context (i.e., perceived pressure to breastfeed and perceived quality of health care professional support), infant feeding method and post‐natal emotional well‐being (i.e., guilt, shame, post‐natal anxiety and post‐natal depression scores). Structural equation modelling revealed that the post‐natal social context indeed predicted post‐natal anxiety and depression scores: guilt, but not shame, mediated these pathways via infant feeding method. These identified differences provide empirical support for the transferability of general definitions of guilt (i.e., as remorse for having committed a moral transgression) and shame (i.e., internalisation of transgressive remorse to the self), to an infant feeding context and provides recommendations for health care practitioners and the maternal social support network to advocate breastfeeding and emotional well‐being outcomes.

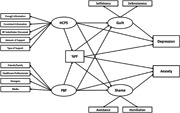

## 1 INTRODUCTION

Exclusive breastfeeding is defined as the administration of solely breast milk to one's infant, without any other foods or liquids, including water (World Health Organisation [WHO], n.d.). Due to the association between breastfeeding and positive maternal and infant health outcomes (Victora et al., 2016) the WHO recommend exclusive breastfeeding for the first 6 months postpartum, and complementary breastfeeding to 2 years and beyond (United Nations International Children's Emergency Fund [UNICEF], 2017). Despite high breastfeeding intention and initiation rates, WHO recommendations are poorly followed in developed countries (McAndrew et al., 2012), and identifying breastfeeding barriers in these countries would help to reduce this initiation‐continuation gap.

Breastfeeding education, promotion and training resources frequently portray breastfeeding as *‘*natural*’* and indicative of *‘*good mothering*’* (Faircloth, 2017; Knaak, 2010). Creating such a moral imperative to breastfeed infers formula supplementation to be an inadequate alternative (Wiant Cummins, 2020), and increases risk of internalised moral sanctioning (Murphy, 1999; Smyth, 2020). For women who do not or cannot breastfeed, this leads to ideal‐actual discrepancy, which positively predicts post‐natal guilt, shame, depression and anxiety (Fallon, Groves, et al., 2016; Lee, 2021; Sonnenburg & Miller, 2021; Taylor & Wallace, 2012). This is problematic because perinatal depression and anxiety cost ~£6.6 billion per 1‐year UK birth cohort (Bauer et al., 2016). High perinatal depression and anxiety also have adverse physical and psychosocial outcomes, such as: increased likelihood of birth complications, lower infant birth weight, lower Apgar score, shorter gestation and longer hospital stay (Dowse et al., 2020); negative impacts on mother‐infant bonding (Davies et al., 2021; Della Vedova et al., 2023); increased likelihood of infant insecure attachment (Barnes & Theule, 2019); shorter breastfeeding duration (Paul et al., 2013); and excessive infant crying (Petzoldt, 2018).

Modifying factors known to contribute toward common aspects of post‐natal distress, such as depression and anxiety (Caldwell et al., 2021), may also offer utility for improving breastfeeding (Dias & Figueiredo, 2015; Fallon, Groves, et al., 2016) and infant development outcomes (Field, 2018; Murray et al., 2018). Guilt and shame have been conceptualised as transdiagnostic phenomena: both underlying the development and/or maintenance of numerous psychological symptoms and disorders (Dalgleish et al., 2020), identifying them as opportune, modifiable factors. Guilt and shame are categorised by remorse, self‐criticism and distress (Tangney & Tracy, 2012; Tangney et al., 2007), which are felt in response to having committed a moral transgression (Tangney & Dearing, 2003).

For guilt, negative emotions are directed toward the moral wrongdoing, while for shame, negative emotions are directed toward the self (Lazare, 1987; Miceli & Castelfranchi, 2018; Tangney et al., 1996). Shame is also more concerned with having failed in front of judging *‘*others*’*, unlike guilt, which is more behaviour orientated (Taylor & Wallace, 2012). Consequentially, guilt is considered more adaptive, due to its associated agency in taking reparative action (Arimitsu, 2001; Lutwak & Ferrari, 1996), while, evolutionarily, fears of experiencing shame are seen as acting as a deterrent from actions which would risk social exile (Crozier, 1998; Gilbert, 2003). In the current study, guilt is defined as, ‘[feeling]…consumed with the idea that they did a “bad thing” (or failed to do a good thing)’ (Niedenthal et al., 1994, p. 587). Similarly, shame is defined as, ‘…an evaluation of the self. Although a specific failure or transgression may trigger a shame reaction, the implications of the event are attributed to the self’. (Niedenthal et al., 1994, p. 586). Previous research has endeavoured to map these general conceptualisations of guilt and shame to the context of infant feeding (Leeming et al., 2021; Thomson et al., 2015). However, these previous endeavours have not produced academic definitions which contain conceptual antecedents and consequences, which can then be quantitatively modelled (Walker & Avant, 2019). A recent mixed methods systematic review found only two studies which had defined guilt and shame in their investigation on infant feeding outcomes, and none had used context‐specific definitions and/or psychometric measures (Jackson et al., 2021a). This is problematic because for other emotional states e.g., anxiety, postpartum‐specific measurement explains unique variance in various health outcomes when compared with general measurement (Fallon, Groves, et al., 2016; Fallon, Halford et al., 2016, 2017).

Jackson et al. (2021b) endeavoured to address this research gap, and identified: pressure to breastfeed (from sources such as media promotion of breastfeeding, sociocultural norms, health care professionals and maternal support networks) and poor perceived quality of health care professional support regarding infant feeding (i.e., having received insufficient information, inconsistent information, a poor amount of support, inappropriate type of support for maternal needs and feeling unable to discuss breast milk substitutes) to be sources of guilt and shame. To date, however, there is an absence of quantitative research which has provided empirical evidence for these theorised relationships, alongside guilt‐specific (i.e., selfishness, defensiveness), shame‐specific (i.e., avoidance behaviour, humiliation) and affective (i.e., post‐natal depression and anxiety scores) consequences (Jackson et al., 2021b).

The current study aims to quantitatively test the validity of aforementioned, theoretically proposed relationships. First, the current study predicted that poorer perceived quality of health care professional support and higher perceived pressure to breastfeed would significantly predict higher formula feeding exclusivity, respective to breastfeeding, and post‐natal anxiety and depression scores. Guilt and shame were expected to mediate the relationship between pressure to breastfeed, perceived quality of health care professional support and post‐natal depression and anxiety scores. Finally, guilt, but not shame, was predicted to significantly mediate the relationship between pressure to breastfeed, perceived quality of health care professional support, infant feeding method, and post‐natal depression and anxiety scores, due to its behaviour‐orientated nature (Niedenthal et al., 1994).

## 2 METHODS

### 2.1 Variable selection

Guilt‐specific consequences (i.e., feeling the need to defend one's infant feeding method, feeling selfish due to one's infant feeding method), and shame‐specific (i.e., feeling the need to avoid certain situations due to one's infant feeding method, feeling humiliated due to one's infant feeding method), were selected based on a synthesis of 20 perinatal papers: which have suggested these variables to be intermediaries between one's social context, infant feeding method and adverse emotional and behavioural outcomes (Jackson et al., 2021b). Although post‐natal anxiety (encompassing panic/fear, and dissociation from one's maternal identity) and depression were suggested to be shame‐specific consequences in supporting theoretical work (Jackson et al., 2021b), there is a strong evidence base for the predictive power of both guilt and shame as determinants of post‐natal anxiety and depression scores (Caldwell et al., 2021; Cândea & Szentagotai‐Tătar, 2018). As such, post‐natal depression and post‐natal anxiety were included as dependent variables of both guilt and shame in the final model.

### 2.2 Participants

Participants self‐selected for the current online survey study through social media platforms e.g., Facebook, Twitter, Instagram and via snowballing. Advertisements were placed in parenting‐specific groups and with parenting specific hashtags to increase visibility. No particular developed countries were targeted for recruitment. The study advertisement, information sheet and consent form merely specified that one must reside in a developed country to take part (United Nations, 2020). Remaining eligibility criteria were as follows: maternal age >18, English‐fluent, with a full term (>37 weeks) infant aged ≤6 months, with a birth weight >2500 g, and without maternal or infant congenital abnormalities contraindicative of breastfeeding.

An a‐priori sample size calculation specified that 799 participants were required, based on: a small‐medium effect size, *d* = 0.15, 0.9 statistical power, and α = 0.05, with four latent variables and 22 observed variables (Christopher Westland, 2010). The first study advertisement was posted on 24 February 2020. The first author checked the progress of survey completions on a weekly basis through Qualtrics. By the 30 March 2020, 1217 post‐natal women had started the questionnaire and 876 had completed it (28.02% attrition). As this value exceeded the a‐priori sample requirement, the survey was closed on 30 March 2020 for analysis.

### 2.3 Measures

The study survey was generated through the Qualtrics online platform. Infant feeding intention, initiation and current method were assessed using 7‐point Likert‐scale response options (Fallon et al., 2017; Table 1 shows response options and frequencies).

**Table 1:** Demographic characteristics of the current sample.

Variable	Category	Frequency (%)
Maternal age	Range	M (SD)
	18–42	30.27 (4.76)
Country of residence	UK	56.4
	USA	29.8
	Canada	4.0
	Other non‐European country	3.2
	Other, European country	2.7
	Ireland	2.7
	Australia	0.9
	New Zealand	0.2
Education level	Undergraduate level or above	72.2
	Other	27.8
Relationship status	Partnered	96.8
	Not partnered	3.2
Employment status	In paid employment	80.8
	Not in paid employment	19.2
Annual household income	£0–£9999	0.8
	£10,000–£19,999	5.8
	£20,000–£29,999	9.3
	£30,000–£39,999	13.1
	£40,000–£49,999	15.6
	£50,000 and over	55.4
Infant feeding intention	Exclusive breastfeeding (100%)	78.7
	Predominantly breast milk (over 80%) with a little formula (under 20%)	10.2
	Mainly breast milk (50%–80%) with some formula	2.9
	A combination of both breast milk (50%) and formula (50%)	3.7
	Mainly formula (50%–80%) with some breast milk	1.0
	Predominantly formula (over 80%) with a little breast milk (under 20%)	0.8
	Exclusive formula feeding (100%)	2.7
Infant feeding initiation	Exclusive breastfeeding (100%)	71.6
	Predominantly breast milk (over 80%) with a little formula (under 20%)	12.0
	Mainly breast milk (50%–80%) with some formula	2.5
	A combination of both breast milk (50%) and formula (50%)	4.6
	Mainly formula (50%–80%) with some breast milk	2.3
	Predominantly formula (over 80%) with a little breast milk (under 20%)	3.0
	Exclusive formula feeding (100%)	4.0
Current infant feeding method	Exclusive breastfeeding (100%)	60.5
	Predominantly breast milk (over 80%) with a little formula (under 20%)	11.3
	Mainly breast milk (50%–80%) with some formula	3.2
	A combination of both breast milk (50%) and formula (50%)	3.1
	Mainly formula (50%–80%) with some breast milk	1.9
	Predominantly formula (over 80%) with a little breast milk (under 20%)	1.6
	Exclusive formula feeding (100%)	18.3
Infant age/weeks by infant feeding method	Exclusive breastfeeding (100%)	14.62 (7.18)
	Predominantly breast milk (over 80%) with a little formula (under 20%)	13.61 (7.54)
	Mainly breast milk (50%–80%) with some formula	12.64 (7.53)
	A combination of both breast milk (50%) and formula (50%)	15.33 (7.75)
	Mainly formula (50%–80%) with some breast milk	16.76 (6.16)
	Predominantly formula (over 80%) with a little breast milk (under 20%)	15.36 (8.63)
	Exclusive formula feeding (100%)	16.98 (7.53)

Theoretically driven (Jackson et al., 2021b) questions were created to assess: perceived quality of infant‐feeding‐related health care professional support, that is, probing perceived satisfaction with amount, type, and consistency of infant feeding information received and perceived ability to discuss formula feeding; perceived pressure to breastfeed, that is, as perceived originating from friends and family, health care professionals, strangers and media. These variables were measured using Likert‐scale response options from 1 ‘Completely disagree’ to 10 ‘Completely agree’, with higher scores representing greater perceived health care professional support quality and greater perceived pressure to breastfeed.

Based on previous literature (Jackson et al., 2021b), guilt was predicted to be associated to feelings of defensiveness and selfishness, due to one's current infant feeding method, while feelings of humiliation and avoidance, again related to one's current infant feeding method, were predicted to be associated to feelings of shame. Specific items were thus created to probe feeling of defensiveness, selfishness, feeling the need to avoid certain situations and feeling humiliated, as a consequence of one's current infant feeding method. Again, Likert‐scale response options from 1 ‘Completely disagree’ to 10 ‘Completely agree’ were used, with higher scores representing stronger defensiveness, selfishness, humiliation feelings; and greater perceived need to avoid certain situations due to one's current infant feeding method.

Post‐natal‐specific anxiety symptoms were measured using the Postpartum Specific Anxiety Scale (PSAS; Fallon, Halford, et al., 2016), which assesses these symptoms as experienced in the last week, using 51‐items across four parenting domains (i.e., competence and attachment, infant safety and well‐being, practical infant care and psychosocial adjustment to motherhood). This measure produces scores ranging from 51 to 204 (with higher scores representing more severe post‐natal‐specific anxiety symptoms), and has been shown to have good convergent validity and excellent test–retest reliability (Fallon, Halford, et al., 2016).

Post‐natal‐specific depression symptoms were measured using the Edinburgh Post‐natal Depression Scale (EPDS; Cox et al., 1987), which probes these symptoms as experienced during the last week, using 10‐items producing final scores ranging 0–30, with higher scores indicating more severe post‐natal‐specific depression symptoms. This questionnaire is the most widely used measure of post‐natal depressive symptoms, and has be shown to have excellent test–retest reliability (Kernot et al., 2015).

### 2.4 Procedure

Ethics approval was obtained from The University of Liverpool ethics committee on 17 February 2020 (Review reference ID number: 6139).

The study was conducted online, with participants able to access it on the Qualtrics.com website. After providing informed consent, participants completed, in order, questions on demographics, formula feeding exclusivity (intention, initiation and current method), health care professional support, pressure to breastfeed, guilt and shame. Participants were then asked to complete the PSAS, and the EPDS.

## 3 DATA ANALYSIS

The number of factors underlying defensiveness, selfishness, avoidance and humiliation were determined using parallel analysis (Hayton et al., 2004). A factor analysis (oblimin rotation) followed, to determine factor loadings. This was conducted to probe the robustness of theoretically driven conceptualisations of guilt and shame (Jackson et al., 2021b) in the current data set, to provide empirical evidence for theoretically proposed distinctions.

**Figure 1:**
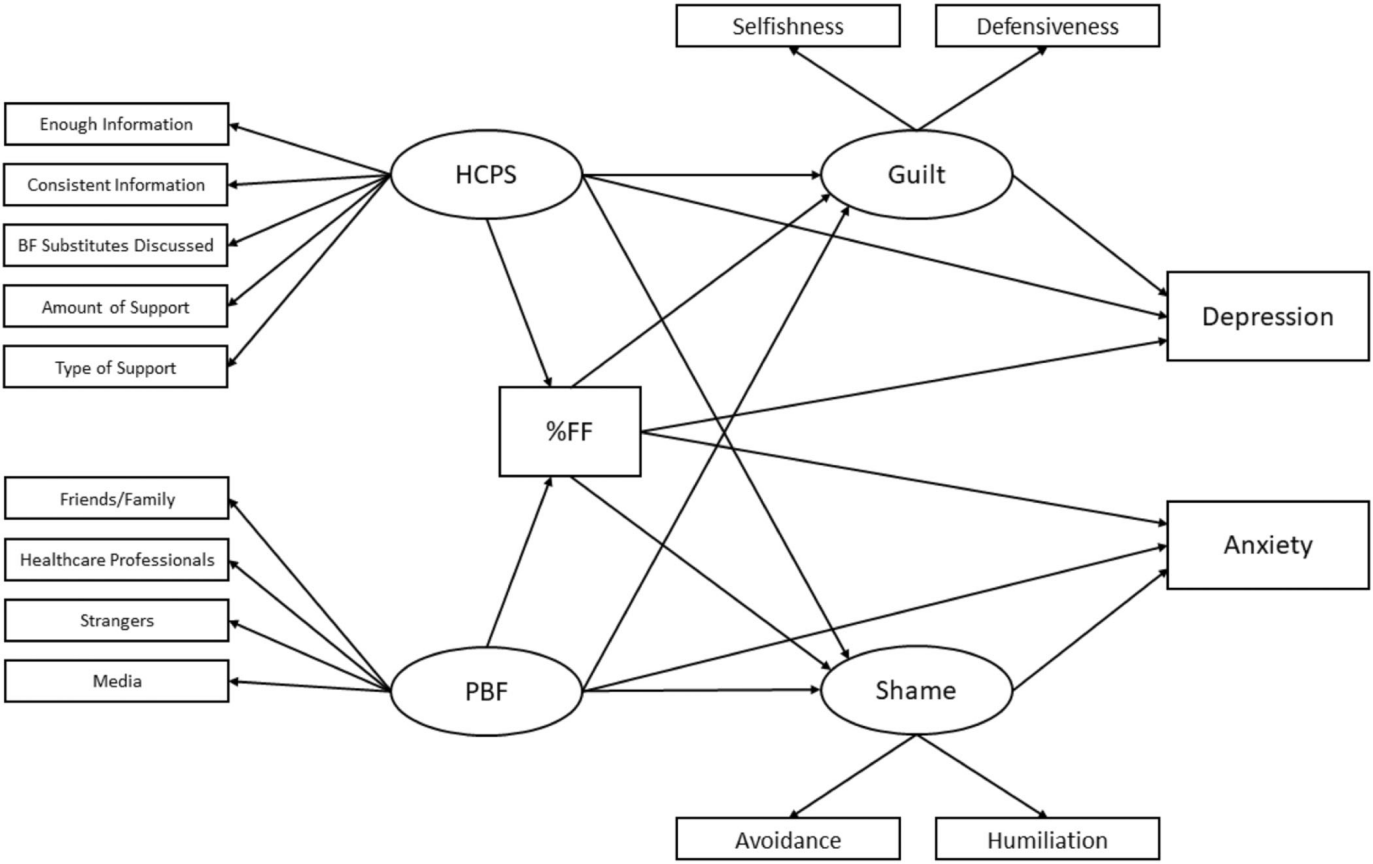
Graphical representation of tested relationships and pathways in the final SEM model. Demographic variables included in the final model tested are reported in the text, but omitted here, for readability purposes. BF, breastfeeding; HCPS, health care professional support; PBF, pressure to breastfeed; %FF, percentage of formula feeding use, respective to breastfeeding.

Structural Equation Modelling (SEM), with Maximum likelihood estimation, tested hypothesised relationships (Figure 1). To maximise model parsimoniousness, demographic variables to retain were identified using stepwise model selection by Akaike Information Criteria (AIC), predicting formula feeding exclusivity, depression and anxiety from demographic variables (initially selected for their influence on breastfeeding practice; McAndrew et al., 2012). Standardised Root Mean Residual (SRMR) absolute fit index, non‐centrality‐based indices, Comparative Fit Index (CFI), Tucker‐Lewis Index (TLI) and Root Mean Square Error of Approximation (RMSEA) estimates were calculated to assess model fit. Indirect effects used bias corrected bootstrap (5000 resamples), and 95% confidence intervals are reported.

## 4 RESULTS

### 4.1 Sample characteristics

Participants were aged 18–42 (Mean 30.27, SD 4.76). Most participants were from the United Kingdom (56.4%) or from United States of America (29.8%). Survey completers were largely undergraduate educated or above (72.1%), partnered (96.8%), in paid employment (80.7%) and from annual income households of >£50,000/annum (55.4%). Regarding infant feeding method, 60.6% of mothers were exclusively breastfeeding, 21.1% of mothers were combination feeding, and 18.3% of women were exclusively formula feeding at the time of investigation. Mean (+SD) infant age in weeks was 14.62 (7.72) for exclusive breastfeeding mothers and 16.98 (7.53) for exclusive formula feeding mothers. See Table 1 for participant demographic characteristics.

Table 2 shows the demographic variables retained in the final SEM. Predictors were retained in the final model if they were found to have a significant effect on formula feeding exclusivity, depression or anxiety, or if their exclusion increased AIC values in the stepwise models.

**Table 2:** Demographic variables retained as predictors of feeding method, depression and anxiety, following stepwise model selection by AIC.

	Feeding method			Depression			Anxiety		
Predictor	*b*	95% CI [LL, UL]	*p* Value	*b*	95% CI [LL, UL]	*p* Value	*b*	95% CI [LL, UL]	*p* Value
(Intercept)	0.960	[0.356, 1.565]	0.002	16.740	[14.115, 19.365]	<0.001	144.301	[131.910, 156.692]	<0.001
Number of children	−0.173	[−0.353, 0.008]	0.060	−0.365	[−0.868, 0.137]	0.154	−7.020	[−9.457, −4.583]	<0.001
Youngest infant age	0.028	[0.011, 0.046]	0.002	0.061	[0.013, 0.110]	0.013	0.322	[0.091, 0.554]	0.006
Feeding intention	0.454	[0.332, 0.576]	<0.001						
Feeding initiation	0.435	[0.336, 0.533]	<0.001						
Maternal age				−0.107	[−0.197, −0.017]	0.020	−0.513	[−0.928, −0.098]	0.015
Income				−0.737	[−1.052, −0.422]		−01.56	[−3.044, −0.075]	0.039
Maternal education	−0.533	[−0.840, −0.227]	0.001	−0.861	[−1.794, 0.071]	0.070			
Maternal occupation	0.403	[0.049, 0.758]	0.026				−4.826	[−9.650, −0.002]	0.050

*Note*: *b* represents unstandardized regression weights. LL and UL indicate the lower and upper limits of a confidence interval, respectively.

### 4.2 Guilt and shame

Parallel analysis indicated two factors underlying defensiveness, selfishness, avoidance and humiliation. Confirming predicted associations, factor analysis found selfishness (0.679) and defensiveness (0.612) loading onto one factor (labelled ‘Guilt’), while avoidance (0.681) and humiliation (0.593) loaded onto the second factor (labelled ‘Shame’), explaining 45.74% of variance. Chosen labels followed relationships predicted in literature (Jackson et al., 2021a, 2021b).

## 5 STRUCTURAL EQUATION MODELLING

The model fit the data well (SRMR = 0.059; RMSEA = 0.058; CFI = 0.925; TLI = 0.908). Table 3 shows SEM latent variable loadings. Items investigating perceived health care professional support quality and pressure to breastfeed were predicted by their relevant latent variable (all *p*s < 0.001). The guilt latent variable predicted selfishness and defensiveness, and the shame latent variable predicted need for avoidance and feelings of humiliation (all *p*s < 0.001).

**Table 3:** Latent variables for pressure to breastfeeding pressure (PBF), health care professional support (HCPS), guilt and shame.

Latent variable	Observed variable	*B*	95% CI [LL, UL]	*p* Value
PBF	Friends/family	1		
	Health care professionals	1.026	[0.894, 1.178]	<0.001
	Strangers	1.208	[1.075, 1.360]	<0.001
	Media	1.230	[1.069, 1.427]	<0.001
HCPS	Enough information	1		
	Consistent information	0.999	[0.938, 1.063]	<0.001
	BF substitutes discussed	0.736	[0.650, 0.824]	<0.001
	Amount of support	1.271	[1.194, 1.364]	<0.001
	Type of support	1.256	[1.180, 1.351]	<0.001
Guilt	Defensiveness	1		
	Selfishness	1.045	[0.908, 1.197]	<0.001
Shame	Avoidance	1		
	Humiliation	1.327	[1.101, 1.627]	<0.001

Table 4 shows the direct and indirect effects tested in the model. The effects on formula feeding exclusivity, anxiety and depression of demographic variables retained in the model fell broadly in line with the results of the stepwise model reported in Table 1.

**Table 4:** Direct and indirect effects.

			Indirect effect			Direct effect		Total effect	
				*95% CI bounds*					
Predictor	Outcome	Mediator(s)	b(SE)	*Lower*	*Upper*	b(SE)	*p* Value	b(SE)	*p* Value
Guilt	Anxiety							5.834(1.422)	<0.001
	Depression							0.927(0.292)	0.001
Shame	Anxiety							6.003(1.625)	<0.001
	Depression							1.102(0.322)	0.001
Formula	Guilt							0.367(0.040)	<0.001
	Shame							−0.014(0.039)	0.726
	Anxiety	Total indirect	2.057(0.663)	0.921	3.540	−2.895(0.704)	<0.001	−0.838(0.449)	0.062
		Guilt	2.138(0.570)	1.225	3.465				
		Shame	−0.081(0.251)	−0.665	0.308				
	Depression	Total indirect	0.325(0.125)	0.101	0.601	−0.301(0.145)	0.035	0.024(0.098)	0.803
		Guilt	0.340(0.113)	0.155	0.594				
		Shame	−0.015(0.045)	−0.122	0.058				
PBF	Formula							0.342(0.057)	<0.001
	Guilt	Formula	0.126(0.022)	0.087	0.176	0.555(0.078)	<0.001	0.681(0.081)	<0.001
	Shame	Formula	−0.005(0.014)	−0.037	0.018	0.682(0.081)	<0.001	0.677(0.072)	<0.001
	Anxiety	Total indirect	7.047(1.673)	4.473	10.943	−2.601(1.757)	0.139	4.446(0.641)	<0.001
		Formula	−0.991(0.302)	−1.674	−0.501				
		Guilt	3.240(0.953)	1.875	5.475				
		Shame	4.094(1.311)	2.099	7.282				
		Formula‐>guilt	0.732(0.230)	0.390	1.307				
		Formula‐>shame	−0.028(0.090)	−0.252	0.101				
	Depression	Total indirect	1.275(0.319)	0.780	1.990	−0.737(0.346)	0.033	0.538(0.134)	<0.001
		Formula	−0.103(0.053)	−0.220	−0.010				
		Guilt	0.515(0.187)	0.235	0.952				
		Shame	0.751(0.250)	0.375	1.362				
		Formula‐>guilt	0.116(0.043)	0.051	0.219				
		Formula‐>shame	−0.005(0.016)	−0.045	0.019				
HCPS	Formula							−0.094(0.035)	0.008
	Guilt	Formula	−0.034(0.013)	−0.064	−0.011	−0.218(0.042)	<0.001	−0.253(0.044)	<0.001
	Shame	Formula	0.001(0.004)	−0.005	0.011	−0.152(0.036)	<0.001	−0.151(0.035)	<0.001
	Anxiety	Total indirect	−2.109(0.566)	−3.399	−1.184	−0.927(0.632)	0.142	−3.036(0.416)	<0.001
		Formula	0.271(0.126)	0.079	0.585				
		Guilt	−1.273(0.391)	−2.274	−0.689				
		Shame	−0.914(0.363)	−1.891	−0.404				
		Formula‐>guilt	−0.200(0.096)	−0.456	−0.059				
		Formula‐>shame	0.008(0.025)	−0.030	0.077				
	Depression	Total indirect	−0.372(0.103)	−0.609	−0.209	−0.173(0.130)	0.183	−0.545(0.092)	<0.001
		Formula	0.028(0.018)	0.003	0.079				
		Guilt	−0.202(0.075)	−0.386	−0.090				
		Shame	−0.168(0.066)	−0.344	−0.071				
		Formula‐>guilt	−0.032(0.017)	−0.079	−0.009				
		Formula‐>shame	0.001(0.005)	−0.005	0.014				
Number of children	Formula							−0.021(0.092)	0.822
	Anxiety							−4.557(1.038)	<0.001
	Depression							−0.001(0.226)	0.996
Youngest infant age	Formula							0.024(0.008)	0.004
	Anxiety							0.222(0.104)	0.032
	Depression							0.040(0.023)	0.083
Feeding intention	Formula							0.419(0.073)	<0.001
Feeding initiation	Formula							0.364(0.060)	<0.001
Maternal age	Anxiety							−0.252(0.196)	0.198
	Depression							−0.080(0.046)	0.083
Income	Anxiety							−1.813(0.701)	0.010
	Depression							−0.747(0.156)	<0.001
Maternal education	Formula							−0.542(0.160)	0.001
	Depression							−0.414(0.329)	0.208
Maternal occupation	Formula							0.467(0.167)	0.005
	Anxiety							−2.896(1.626)	0.075

Abbreviations: b, unstandardized regression weights; CI, confidence interval; SE, standard errors.

### 5.1 Formula feeding exclusivity effects on post‐natal depression and post‐natal anxiety, as mediated by guilt and shame

As shown in Table 4, both guilt and shame were significantly and positively associated with both post‐natal anxiety and post‐natal depression. The degree of formula feeding exclusivity was also positively associated with guilt.

While the total effect of formula feeding exclusivity on post‐natal anxiety and post‐natal depression was nonsignificant, a pattern of inconsistent mediation (Hayes, 2009; MacKinnon et al., 2002) was evident: an increase in the degree of formula feeding exclusivity led to significantly decreasing anxiety and depression, but this was counterbalanced by the fact that increasing formula feeding exclusivity increased guilt, which ultimately led to an increase in post‐natal anxiety and post‐natal depression. No mediation through shame was found.

### 5.2 Pressure to breastfeed effects on post‐natal anxiety and post‐natal depression, as mediated by formula feeding exclusivity, guilt and shame

As also shown in Table 4, increasing pressure to breastfeed predicted an increase in guilt, shame and the degree of formula feeding exclusivity. Only for guilt, an indirect effect through formula feeding exclusivity was found (explaining 18.50% of pressure to breastfeed effects on guilt, which however remained significant when accounting for this mediation): the increase in pressure led to an increase in the degree of formula feeding exclusivity, which ultimately increased guilt.

Pressure to breastfeed was also positively associated with post‐natal anxiety, although this effect became nonsignificant when accounting for indirect pathways. Guilt and shame were significant mediators, explaining 27.73% and 35.03%, respectively, of pressure to breastfeed effects on post‐natal anxiety. Increasing pressure to breastfeed increased guilt and shame, which led to an increase in post‐natal anxiety. The indirect effect through formula feeding exclusivity (which explained 8.48% of pressure to breastfeed effects on post‐natal anxiety) had opposite sign to the total effect of pressure to breastfeed on post‐natal anxiety: while greater pressure to breastfeed increased formula feeding exclusivity, higher levels of the latter decreased post‐natal anxiety. This potentially protective effect was mostly nullified by a multiple mediation through formula feeding exclusivity and guilt (explaining 6.26% of pressure to breastfeed effects on post‐natal anxiety): greater pressure to breastfeed increased formula feeding exclusivity, which increased guilt, which in turn increased post‐natal anxiety. No similar multiple mediation through formula feeding exclusivity and shame was found.

Finally, pressure to breastfeed was also positively associated with post‐natal depression. The pattern of indirect effects leading to depression was found to be broadly similar to those evidenced for anxiety. Guilt, shame and formula feeding exclusivity explained 23.13%, 33.72% and 4.63%, respectively, of pressure to breastfeed effects on post‐natal depression. A multiple mediation through formula feeding exclusivity and guilt (explaining 5.21% of pressure to breastfeed effects on post‐natal depression) overwhelmingly compensated for the pathway from pressure to breastfeed to post‐natal depression, through formula feeding. Notably, when accounting for these indirect pathways, the effect of pressure to breastfeed on depression remained significant but switched sign: increasing pressure to breastfeed appeared to decrease post‐natal depression.

### 5.3 Health care professional support effects on post‐natal anxiety and post‐natal depression, as mediated by formula feeding exclusivity, guilt and shame

As shown in Table 4, health care professional support was found to be negatively associated with the degree of formula feeding exclusivity, and with guilt and shame. Only for guilt, an indirect effect through formula feeding exclusivity was found (explaining 13.44% of health care professional support effects, which remained significant when accounting for this mediation), with increasing support leading to a decrease in formula feeding exclusivity and ultimately a decrease in guilt.

Health care professional support was also negatively associated with post‐natal anxiety. This effect became nonsignificant, however, when accounting for indirect pathways. Guilt and shame were significant mediators, explaining 35.43% and 25.44%, respectively, of health care professional support effects on post‐natal anxiety: increasing health care professional support decreased guilt and shame, and, consequently, post‐natal anxiety. The indirect effect through formula feeding exclusivity (which explained 7.54% of health care professional support effects on post‐natal anxiety) had opposite sign to the total effect of health care professional support on post‐natal anxiety: while greater health care professional support decreased formula feeding exclusivity, lower levels of the latter increased post‐natal anxiety. This effect was largely buffered by a multiple mediation through formula feeding exclusivity and guilt (explaining 5.57% of health care professional support effects on post‐natal anxiety): greater health care professional support decreased formula feeding exclusivity, which decreased guilt, which in turn decreased post‐natal anxiety. No similar multiple mediation through formula feeding exclusivity and shame was found.

Finally, health care professional support was also negatively associated with post‐natal depression, but this effect became nonsignificant when accounting for indirect effects, which showed similar patterns to those for post‐natal anxiety. Guilt, shame and formula feeding exclusivity explained 33.44%, 27.81% and 4.64%, respectively, of health care professional support effects on post‐natal depression. A multiple mediation through formula feeding exclusivity and guilt (explaining 5.30% of health care professional support effects on post‐natal depression) overwhelmingly compensated for the pathway from health care professional support to post‐natal depression, through formula feeding exclusivity.

## 6 DISCUSSION

The current study aimed to provide empirical evidence for the theoretically proposed relationships between perceived pressure to breastfeed, quality of health care professional support, infant feeding method, guilt, shame and post‐natal depression and anxiety. This supports concept exclusivity and substantiates pre‐existing definitions of guilt and shame to an infant feeding context (Gilbert, 2003; Jackson et al., 2021b; Leeming et al., 2021; Lutwak & Ferrari, 1996; Niedenthal et al., 1994; Tangney et al., 1996; Taylor & Wallace, 2012; Thomson et al., 2015). Shame was not affected by formula feeding exclusivity (a maternal enacted behaviour) but was rather a product of maternal social context forces (i.e., health care professional support and pressure to breastfeed), explaining much of their effects on post‐natal maternal mental health. Maternal behaviour (i.e., formula feeding exclusivity), significantly affected by the social context, led instead to guilt and consequently poorer post‐natal mental health. Guilt was further supported, therefore, as a transgression‐orientated emotion (e.g., Niedenthal et al., 1994; Tangney et al., 1996).

Breastfeeding is often synonymised with *‘*good mothering*’* in promotional discourse (Marshall et al., 2007). In the current study, transgressing this perceived standard indirectly increased depression and anxiety scores. This relationship is supported by pre‐existing theory, with internalised ideal‐actual discrepancy resulting in moral sanctioning of the self (Lee, 2021; Murphy, 1999; Smyth, 2020; Sonnenburg & Miller, 2021; Taylor & Wallace, 2012). Shame, instead, concerns self‐perceptions of inadequacy, within one's social context, which is not necessarily tied to behaviour (Crozier, 1998; Gilbert, 2003; Niedenthal et al., 1994; Sonnenburg & Miller, 2021; Taylor & Wallace, 2012). This indirectly increased anxiety and depression scores, which may by motivated by fears of judgement and associated help‐seeking avoidance (Appleton et al., 2018; Dunford & Granger, 2017).

Guilt and shame both contributed toward post‐natal anxiety and depression scores, which supports their conceptualisation as transdiagnostic phenomena (Dalgleish et al., 2020). A recent systematic review also found that both concepts are significantly related to post‐natal depression, though the effects of guilt were reduced when including those of shame (Caldwell et al., 2021). Recent works suggest this might also be a function of self‐discrepancy theory: shame predisposes individuals to experience depression because both emotions are characterised by high ideal‐actual discrepancy, and by tendencies to ruminate and to hold pessimistic, stable, global self‐directed attributions (Sonnenburg & Miller, 2021). Routine mental health screening in maternity care is often informal and lacking in cohesiveness, which can result in maternal needs going unmet (Mellor et al., 2019). For example, midwives report that for milder‐moderate anxiety and depression, criteria are not sufficiently met for referral to mental health services (Mellor et al., 2019; Ministry of Health, 2012). Rather than solely relying on screening categories as indicators of support need, the current study suggests that removing distinctions between clinical and nonclinical taxa would allow for more comprehensive treatment pathways for post‐natal distress to be achieved (Dalgleish et al., 2020; Mellor et al., 2019).

Furthermore, barriers to self‐disclosure among postpartum women include perceived stigma and shame around experiencing mental health difficulties, and time restraints placed on interactions with health care practitioners (Nagle & Farrelly, 2018). Again, by health care practitioners adopting a more holistic approach to mental health screening, achieved by placing greater onus on perceived level of maternal need and proactive engagement in conversations about milder‐more moderate aspects of mental distress at every routine appointment (opposed to relying exclusively on reductionist screening measures to determine level of mental health need), may ease symptom severity (Caldwell et al., 2021; Cândea & Szentagotai‐Tătar, 2018) and improve satisfaction with quality of mental health care support (Nagle & Farrelly, 2018). This may in turn benefit post‐natal maternal and infant outcomes (Barnes & Theule, 2019; Davies et al., 2021; Dias & Figueiredo, 2015; Fallon, Groves, et al., 2016; Murray et al., 2018; Paul et al., 2013; Vedova et al., 2023).

Interestingly, while formula feeding exclusivity alone was unrelated to post‐natal anxiety and depression, investigating the roles of guilt and shame revealed complex networks of effects (Hayes, 2009; MacKinnon et al., 2002). On the one hand, increasing formula feeding exclusivity directly decreased post‐natal anxiety and depression scores. Breastfeeding challenges, for example, perceived insufficient milk supply and latching difficulties, are common and cause distress regarding infant weight gain (Davie et al., 2021). Current findings suggest that formula milk supplementation may lead to feelings of resolution and relief for these mothers.

However, this effect was counterbalanced by multiple mediations through guilt: increasing formula feeding exclusivity increased guilt, which heightened post‐natal anxiety and depression scores. It is notable that only when removing its perception as transgressive, is formula supplementation alleviating of anxiety and depression. Shame, on the other hand, did not significantly mediate formula feeding exclusivity. Instead, shame directly, positively correlated with post‐natal anxiety and depression, substantiating distinctions between guilt and shame, conceptually (Arimitsu, 2001; Crozier, 1998; Gilbert, 2003; Lazare, 1987; Miceli & Castelfranchi, 2018; Niedenthal et al., 1994; Tangney et al., 1996; Taylor & Wallace, 2012).

Quality of health care professional support was also negatively associated with post‐natal anxiety. This effect became nonsignificant, however, when accounting for indirect pathways. Guilt and shame were significant mediators of health care professional support effects on post‐natal anxiety: increasing health care professional support decreased guilt and shame, and consequently, post‐natal anxiety. While greater health care professional support also decreased formula feeding exclusivity, lower levels of the latter were found to increase post‐natal anxiety, but this was largely buffered by the reduction in guilt brought about by decreased use of formula feeding. Current findings are especially poignant for supporting populations of new mothers who are traditionally less likely to breastfeed e.g., from lower income households, in full‐time employment and single mothers (Hawkins et al., 2007; Ryan et al., 2006; Simpson et al., 2019). Here, high quality health care professional support is highlighted as an instrumental optimiser of breastfeeding and maternal emotional well‐being outcomes.

Pressure to breastfeed was positively associated with post‐natal anxiety, although this effect became nonsignificant when accounting for indirect pathways through guilt, shame and formula feeding exclusivity. Anxiety increased due to higher pressure to breastfeed, which increased shame scores. Restricting conversations about breastfeeding alternatives reaffirms perceived stigma around formula supplementation as *‘*bad’, ‘suboptimal’ or even ‘harmful’ (Marshall et al., 2007). Perceived time restraints on interactions with health care professionals about infant feeding may reiterate beliefs that one's body (i.e., the self) is failing a ‘natural’, ‘good’, and ‘easy’ task, which causes distress (Jackson et al., 2021a). Regarding indirect pathways, pressure to breastfeed increased formula feeding exclusivity, which increased guilt and anxiety scores. Feeling greater pressure to breastfeed from one's social support network indoctrinates a moral imperative to exclusively breastfeed (Murphy, 1999; Smyth, 2020; Wiant Cummins, 2020). This uncompromising mentality, permeating breastfeeding promotion (Fallon et al., 2019), leaves women reluctant to supplement with formula milk when breastfeeding difficulties are faced, which may otherwise have enabled partial breastfeeding continuation. This heightens complete breastfeeding cessation risk in social contexts that dictate exclusive breastfeeding, rather than supporting breastfeeding as a process (Braimoh & Davies, 2014; Brown, 2016; Símonardóttir & Gíslason, 2018).

Removing breastfeeding imperatives from infant feeding discussions, encouraging incremental goal setting and normalising common breastfeeding difficulties during routine care is recommended. These recommendations echo calls to action detailed in pre‐existing infant feeding literature (e.g., Brown et al., 2011; Byrom et al., 2021) and policy documentation (UNICEF, 2022). Standardising perinatal mental health and infant feeding training as routine among health care staff (McLelland et al., 2015; Myors et al., 2012; Webb et al., 2021), fostering staff esteem and resilience to work collaboratively with women and their families during infant feeding discussions (Gustafsson et al., 2017; Maunder et al., 2006), and addressing institutional barriers surrounding insufficient staffing and time restraints (e.g., through active recruitment campaigns into health care career pathways; Brooks et al., 2014), would nurture an environment whereby maternity staff can provide the quality of care preferred among both staff and mothers (Bäckström et al., 2010; Wieczorek et al., 2016).

For new mothers, societal attitudes are perceived to be a major source of perceived pressure to breastfeed (Korth et al., 2022). Health promotional campaigns have shown to be effective in improving mental health literacy and attitudes among the public (Livingston et al., 2013; Thornicroft et al., 2016). Establishing an educational public health campaign which encourages social support networks to become breastfeeding and emotional well‐being advocates may, as such, offer an opportune solution for fostering positive maternal mental health and breastfeeding outcomes.

While pressure to breastfeed increased post‐natal depression, this detrimental effect was entirely explained by indirect influences through formula feeding exclusivity, guilt and shame, in line with what was described for post‐natal anxiety. Experiencing greater pressure to breastfeed may also contribute toward post‐natal depression via ideal‐actual discrepancy associated with perceiving oneself to be failing personal breastfeeding expectations (Lee, 2021; Sonnenburg & Miller, 2021; Taylor & Wallace, 2012). When accounting for indirect influences, the direct effect of pressure to breastfeed on post‐natal depression remained significant but switched sign. Thus, greater pressure to breastfeed appeared to protect against post‐natal depression experience (while nonsignificant, the direct effect of pressure to breastfeed on post‐natal anxiety also switched sign, compared to its total effect). This counterintuitive finding reveals the multifaceted nature of pressure to breastfeed in determining maternal mental well‐being outcomes.

Specifically, the direct effect of pressure to breastfeed on post‐natal depression captures those aspects of pressure to breastfeed centred around the influence that one's social environment has on infant feeding decision making (pressure to breastfeed‐related questions asked about family, friends, and health care workers), after separating out its guilt‐ and shame‐inducing effects. This depression‐lowering direct effect is supported by existing infant feeding literature evidencing a positive effect of social support on maternal mental health (Leahy‐Warren et al., 2012; Xie et al., 2009). By contrast, this complex pattern of effects also captures the gravity which guilt‐ and shame‐inducing components of pressure to breastfeed hold on maternal mental well‐being: their overwhelming intensity nullified, nay reversed potentially positive effects of social network involvement on maternal well‐being, within the context of infant feeding decision‐making.

The direct relationships between health care professional support and post‐natal anxiety and depression symptoms were entirely explained by indirect pathways through guilt, shame and formula feeding exclusivity: increased anxiety and depression resulted from poorer health care professional support increasing feelings of guilt and shame, and formula use, which in turn further increased guilt. In existing literature, ineffective health care professional support has been linked with early breastfeeding cessation, guilt, shame (Jackson et al., 2021a), anxiety and depression (Dias & Figueiredo, 2015; Fallon, Groves, et al., 2016). In the current study, poor health care professional support reflected feelings of having received insufficient, inconsistent, and unsatisfactory infant feeding information and feeling unable to discuss formula feeding. Providing balanced, consistent and crucially, non‐judgemental breastfeeding support may therefore allow for health care professionals to be influential facilitators of improved breastfeeding outcomes. Please see Table 5 for a summary of study recommendations.

**Table 5:** Summary of study recommendations for research and for practice.

Recommendation	Details of study recommendation
Health care professionals should adopt a more holistic approach to post‐natal mental health screening during routine care	Rather than solely relying on categorical screening measures as indicators of support need (Mellor et al., 2019; Ministry of Health, 2012), recommendations are made to place greater onus on the perceived mental health needs of the mother. This can be achieved by health care practitioners engaging pro‐actively in conversations about milder aspects of emotional distress at each routine appointment and to decide collaboratively with the mother whether additional support would be of benefit.
Health care professionals should be open to conversations about safe formula milk supplementation, to dissipate moral imperatives to breastfeed exclusively	Impeding conversations about formula milk supplementation reaffirms stigma of its practice being suboptimal (Wiant Cummins, 2020). Removing imperatives to breastfeed exclusively from infant feeding discussions is recommended. This alteration might serve to nurture any breastfeeding continuation for mothers who would otherwise stop breastfeeding altogether when challenges are experienced (e.g., Davie et al., 2021). Encouraging incremental goal setting, normalising common breastfeeding difficulties, and providing balanced and mother‐centred infant feeding options during routine care is also recommended to this avail (e.g., Brown, 2016). High quality health care professional support protects maternal emotional wellbeing and optimises breastfeeding outcomes. This is especially important for encouraging new mothers who are traditionally less likely to breastfeed (Hawkins et al., 2007; Ryan et al., 2006; Simpson et al., 2019).
Infrastructure in health care settings needs to be strengthened to enable practitioners to provide quality of infant feeding and emotional well‐being care which is preferred by staff and mothers	Standardising perinatal mental health and infant feeding training as routine among health care staff (McLelland et al., 2015; Myors et al., 2012; Webb et al., 2021), fostering staff esteem and resilience to work collaboratively with women and their families during infant feeding discussions (Gustafsson et al., 2017; Maunder et al., 2006), and addressing institutional barriers surrounding insufficient staffing and time restraints (e.g., through active recruitment campaigns into health care career pathways; Brooks Carthon et al., 2014), would rear an environment whereby maternity staff can provide the quality of care preferred by both staff and mothers (Bäckström et al., 2010; Wieczorek et al., 2016)
A health promotion campaign should be developed and implemented to raise awareness, educate and improve societal attitudes toward maternal mental health and breastfeeding difficulties	Societal attitudes are a major source of pressure to breastfeed (Korth et al., 2022). Health promotional campaigns have shown to be effective in improving mental health literacy and attitudes among the public (Livingston et al., 2013; Thornicroft et al., 2016). Establishing an educational public health campaign which encourages social support networks to become advocates for breastfeeding and post‐natal emotional well‐being is thus recommended (Leahy‐Warren et al., 2012; Xie et al., 2009). Supporting breastfeeding as a process, too, might prove fruitful for optimising breastfeeding continuation (Braimoh & Davies, 2014; Brown, 2016; Símonardóttir & Gíslason, 2018).
Guilt and shame are nuanced, but distinct emotions and should be investigated from this standpoint in perinatal research	Shame was not affected by infant feeding method, unlike guilt. Instead, shame was the product of maternal social context forces. Although guilt and shame both predicted post‐natal anxiety and depression scores, these moral emotions are distinct and should be investigated as separate constructs in perinatal research.

### 6.1 Study limitations

First, most recruited participants were highly educated, partnered, and from annual household incomes of >£50,000, possibly limiting finding generalisability. The current study obtained an especially high percentage of exclusively breastfeeding women (initiation = 71.6%, method at time of investigation = 60.5%). In pre‐existing literature, feelings of guilt magnify with increasing formula milk supplementation (Fallon, Komninuo, et al., 2016; Komninou et al., 2016). Likewise, experience of shame differs by infant feeding method (Hanell, 2017; Thomson et al., 2015). As such, current findings may not be truly exemplary of combination and exclusively formula feeding mothers, restricting application in the broader infant feeding landscape. Additionally, the study was cross‐sectional, thus possibly susceptible to transient confounders, particularly given its focus on mood (Spector, 2019). Future research should longitudinally investigate infant feeding related emotional experiences, to better examine causality.

Infant feeding is complex and determined by a multitude of structural, community and individual level factors (Rollins et al., 2016). The current study did not routinely gather information on factors known to predict infant feeding decision making and experience, such as: parity (Hackman et al., 2015), infant feeding history (Darwent et al., 2016), mode of delivery and birth complications (Brown & Jordan, 2012; Hobbs et al., 2016), challenges to breastfeeding e.g., nipple pain during feeds and infant tongue tie (Mahurin‐Smith, 2023; Schlatter et al., 2019), relationship quality with one's partner (Yan et al., 2023), immigration status and ethnicity (Gibson‐Davis & Brooks‐Gunn, 2006). It should be noted, therefore, that the current study offers a limited view of this multifaceted phenomenon.

Guilt and shame were also measured in a reductionist fashion. To elaborate, these moral emotions elicit behavioural responses (Fahlquist, 2016; Fox et al., 2015; Lee & Furedi, 2005) in accompaniment of emotional characteristics which were recorded in the current investigation. Additionally, guilt and shame are both believed to be associated with the cognitive, ruminating tendencies to engage in counterfactual thinking (Niedenthal et al., 1994). Although the decision to focus on emotional characteristics of guilt and shame was made to provide quantitative evidence for theoretically justified, mapped relationships (Jackson et al., 2021b), not accounting for these broader conceptual components may provide an overly simplified view of guilt and shame.

Breastfeeding initiation is defined as putting one's new‐born to the breast within the first hour of life (World Health Organisation and UNICEF, 2018). However, a definition of breastfeeding initiation was not provided to respondents during survey completion, which may have opened the study to unanticipated variance regarding the interpretation of this key definition. Finally, data collection spanned a period whereby national‐level preventative measures were being emplaced to tackle growing concerns about spread and mortality of the novel COVID‐19 virus (US Department of Defense, 2023; World Health Organisation, 2021). There is a plethora of evidence to suggest that social isolation and anxieties pertaining to the pandemic adversely affected maternal mental health (Diamond et al., 2020; Hessami et al., 2022). Social distancing practices and measurement of anticipatory anxiety were not routinely collected in the current study, and so the authors cannot feasibly estimate the extent to which lockdown‐related, or expectancy‐related stressors may have confounded measures of affect.

## 7 CONCLUSION

The current study aimed to provide empirical evidence for theoretically proposed relationships mapped between perceived pressure to breastfeed, quality of health care professional support, infant feeding method, guilt, shame, and post‐natal depression and anxiety scores. The current model identified a negative relationship between health care professional support, and post‐natal anxiety and depression symptoms, and a positive relationship between pressure to breastfeed and post‐natal anxiety and depression symptoms. These effects were, however, explained by indirect pathways through increases in guilt and shame. Formula feeding exclusivity was negatively correlated with post‐natal anxiety symptoms. Higher formula feeding exclusivity, related to low health care professional support and high pressure to breastfeed, increased guilt score, bringing about an increase in post‐natal depression and anxiety symptoms. Shame related to low health care professional support and high pressure to breastfeed, however, acted on post‐natal depression and anxiety symptoms independently of infant feeding method. This substantiates general definitions of guilt and shame to an infant feeding context.

Recommendations are made for health care professionals to pro‐actively engage postpartum women in conversations about milder‐more moderate aspects of mental distress at routine appointments and to place greater onus on maternal mental health needs, opposed to overly relying on standardised, categorical screening measures of affect to determine levels of need. Providing perinatal mental health training to health care staff would facilitate this suggestion. Other recommendations include encouraging incremental infant feeding goal setting, promoting breastfeeding with use of more emotionally neutral language, and normalising breastfeeding difficulties by national campaigning, to encourage any breastfeeding continuation and support from one's social support network.

## AUTHOR CONTRIBUTIONS

Leanne Jackson was responsible for investigation, and writing, original draft. Leanne Jackson and Leonardo De Pascalis were involved in methodology, data curation, software, formal analysis, validation and visualization. All named authors were involved in study conceptualisation, and writing, review and editing. All named authors reviewed and consented to the final manuscript submission.

## CONFLICT OF INTEREST STATEMENT

The authors declare no conflict of declare.

## Data Availability

The final anonymised data set will be made available via open access publishing and data sharing from the University of Liverpool Repository, through Liverpool Research Data: https://livrepository.liverpool.ac.uk/ in line with Data Sharing and Good Academic Practice Agreements. This is in accordance with the University of Liverpool Open Access Publication Policy, and in line with the Research Excellence Framework policy.
